# Multi-cancer samples clustering via graph regularized low-rank representation method under sparse and symmetric constraints

**DOI:** 10.1186/s12859-019-3231-5

**Published:** 2019-12-30

**Authors:** Juan Wang, Cong-Hai Lu, Jin-Xing Liu, Ling-Yun Dai, Xiang-Zhen Kong

**Affiliations:** School of Information Science and Engineering, Qufu Normal University, Rizhao, China

**Keywords:** Affinity matrix, Gene expression data, Graph regularization, Symmetric constraint, Low-rank representation, Spectral clustering

## Abstract

**Background:**

Identifying different types of cancer based on gene expression data has become hotspot in bioinformatics research. Clustering cancer gene expression data from multiple cancers to their own class is a significance solution. However, the characteristics of high-dimensional and small samples of gene expression data and the noise of the data make data mining and research difficult. Although there are many effective and feasible methods to deal with this problem, the possibility remains that these methods are flawed.

**Results:**

In this paper, we propose the graph regularized low-rank representation under symmetric and sparse constraints (sgLRR) method in which we introduce graph regularization based on manifold learning and symmetric sparse constraints into the traditional low-rank representation (LRR). For the sgLRR method, by means of symmetric constraint and sparse constraint, the effect of raw data noise on low-rank representation is alleviated. Further, sgLRR method preserves the important intrinsic local geometrical structures of the raw data by introducing graph regularization. We apply this method to cluster multi-cancer samples based on gene expression data, which improves the clustering quality. First, the gene expression data are decomposed by sgLRR method. And, a lowest rank representation matrix is obtained, which is symmetric and sparse. Then, an affinity matrix is constructed to perform the multi-cancer sample clustering by using a spectral clustering algorithm, i.e., normalized cuts (Ncuts). Finally, the multi-cancer samples clustering is completed.

**Conclusions:**

A series of comparative experiments demonstrate that the sgLRR method based on low rank representation has a great advantage and remarkable performance in the clustering of multi-cancer samples.

## Background

Currently, cancer is one of the most prevalent human diseases, and cancer seriously threatens the quality of human life [1]. The number of variety cancers is increasing, which makes it difficult to effect a radical cure of cancer. A good performing cancer diagnosis method can help doctors to formulate treatment strategies for patients effectively and in a timely manner. In addition, cancer clustering based on gene expression data has become one of the frontiers of bioinformatics research. In the field, it provides an effective way to further explore gene expression data. For example, it can be used to classify cancer [2], select genes [3] and discover cancer linked biomarker genes [4]. In this paper, we propose a methodology to processing gene expression data for identifying different types of cancer.

Since the start of the twenty-first century, the volume of high dimensional and complex gene expression data has exploded with the advent and development of gene detection technology such as DNA microarray technology [5]. So far, the researchers have proposed many well-performing methods and used them for gene expression data mining, such as K-means clustering [6], nonnegative matrix factorization (NMF) [7] and principal component analysis (PCA) [8]. More recently, because of the high dimensional nature of gene expression data, the low-rank representation (LRR) method has become a popular and promising method since its prototype was proposed by Liu et al. [9]. The LRR method can preserve the subspace structure of the raw dataset in a lowest rank representation matrix. Theoretically, the lowest rank representation matrix is a block-diagonal matrix with a well grouped effect, and this matrix can well capture the global structural information of high-dimensional dataset [10]. And then, the clustering method, such as spectral clustering method, is used to cluster the lowest rank representation matrix to realize the subspace segmentation. The LRR clustering method has been adopted widely in many fields due to the advantages of the lowest rank representation matrix, such as facial recognition [11], genetic microarray data clustering [12], image clustering [13] and subspace segmentation [14]. And, LRR method achieves good results in processing high-dimensional datasets.

In general, high-dimensional data always have noisy and outliers because of the complexity of the collection process. And, the noisy and outliers inevitably impairs the intrinsic structure of the data space. Therefore, the outliers and noise cause difficulties during processing the raw data. Especially in the LRR method, the high-dimensional data are usually used in the form of a dictionary matrix, which inevitably adversely affects grouped effect of lowest rank representation matrix. As described in [9], the LRR method may fail to obtain a block-diagonal lowest rank representation matrix in complex applications, which makes it difficult to integrate the lowest rank representation matrix with other information. To alleviate this problem, the commonly used solution is to combine the LRR method with the spectral clustering method (the Ncuts clustering method is often adopted) to get the final clustering result. The LRR method and the spectral clustering method are linked by an affinity matrix which is constructed based on the lowest rank representation matrix. And, the affinity matrix has better grouping effects. In general, in order to construct the affinity matrix, a symmetric operation step is usually performed to establish a similarity-based undirected graph. However, this simple symmetric operation inevitably leads to the loss of important structural information of the raw dataset. To tackle this disadvantage, Ni et al. proposed an approach named the low-rank representation with positive semi-definite (LRR-PSD) to obtain a symmetric positive semi-definite (PSD) matrix [15]. In the LRR-PSD method, an affinity matrix is constructed based on the PSD matrix. This method inspired Chen et al. to propose a low-rank representation with symmetric constraint (LRRSC) method for learning a symmetric lowest rank representation matrix [16]. In this method, the affinity matrix is constructed according to the angular information of the principal directions of the symmetric lowest rank representation matrix. The obtained affinity matrix is better than the matrix which is obtained by simple symmetric operation.

However, compared with the sparse representation method, which considers the sparsest representation of each data point or data vector individually, the LRR method mainly focuses on the global structural information of data [9]. That leads to the LRR method ignoring the local geometrical structural information of data. Because it is shown that the intrinsic local geometrical structures within the high-dimensional data are important for the subspace clustering model [17], some researchers introduce nonlinear dimensionality reduction methods into the LRR, such as the manifold learning theory.

At present, many well-established nonlinear dimensionality reduction methods have been proposed since Tenenbaum et al. and Roweis et al. proposed isometric mapping (ISOMAP) [18] and locally linear embedding (LLE) [19], respectively. The typical methods include the locality preserving projection (LPP) [20], local tangent space alignment (LTSA) [21], Laplacian eigenmap (LE) [22] and Riemannian normal coordinates (RNC) [23]. They can generate a low-dimensional subspace according to the submanifold of the observation datasets. Furthermore, the manifold learning method treats the local geometrical structures of data points as submanifolds. Inspired by the local invariance [20], the manifold learning method estimates the geometrical structures of the submanifold using random data points [24]. The method can map the submanifolds from the high-dimensional space to the low-dimensional space. Therefore, the local geometrical structural information of the raw high-dimensional dataset can be preserved in the low-dimensional space [25].

In order to improve the original LRR method, some researchers combine manifold learning theory with the LRR. For instance, Yin et al. proposed a novel model called the nonnegative sparse hyper-Laplacian regularized LRR (NSHLRR) that can acquire the inherent information within dataset [24]. Motivated by the NSHLRR, Wang et al. proposed the Laplacian regularized low-rank representation (LLRR) method to identify differently expressed genes [26]. More recently, Wang et al. presented a tumor sample clustering method named the Mixed-norm Laplacian Regularized low-rank representation (MLLRR) [27].

Motivated by the above methods, in order to obtain a better lowest rank matrix that can avoid the simple symmetric operation and preserve the intrinsic local geometrical structures within the raw high-dimensional dataset, we introduce symmetric sparse constraints and graph regularization based on manifold learning into the LRR method, and propose the graph regularized low-rank representation method under combined the sparse and symmetric constraints, or short sgLRR method. The sgLRR method can obtain a lowest rank representation matrix that can well capture the global structure information of the high-dimensional raw dataset and meanwhile preserve the intrinsic local geometrical structures within the dataset. Furthermore, the sgLRR method weakens the adverse effect of noise in the raw dataset by strengthening the symmetric constraint to the lowest rank representation matrix. The obtained lowest rank representation matrix is an excellent basis for constructing the affinity matrix. To take full advantage of the lowest rank representation matrix, we consider the angular information of the principal directions of the lowest rank representation matrix. Therefore, in contrast to the traditional approach, we perform skinny singular value decomposition (SVD) operations on the lowest rank representation to construct the affinity matrix. Finally, based on the affinity matrix, we adopt a spectral clustering algorithm to obtain the clustering results.

We adopt the sgLRR method for multi-cancer sample clustering based on gene expression data. Our experiment design is carried out in the following three steps: Step One: the sgLRR method is used to process multi-cancer sample gene expression dataset. And, we can obtain a lowest rank representation matrix. Step Two: an affinity matrix is constructed by exploiting the obtained lowest rank representation matrix. Step Three: based on the affinity matrix, we adopt a spectral clustering algorithm, i.e., Ncuts method, to perform the multi-cancer sample clustering. Compared with a lot of related methods, the sgLRR method has better performance in multi-cancer sample clustering.

In summary, the main contributions of our work are as follows:

1. We introduce the symmetric sparse constraints and graph regularization based on manifold learning into the original LRR method and develop a novel method named the sgLRR. The regularized graph is better for preserving the local geometrical structure of raw high-dimensional data. And, the symmetric constraint weakens the effect of noise in the raw dataset. Therefore, we use sgLRR method to get a better lowest rank presentation matrix that has better grouping effect for the subspace clustering.
2. Based on the lowest rank presentation matrix, we construct an affinity matrix to further improve its grouping effect. As the link of sgLRR method and Ncuts clustering method, the affinity matrix makes full useful of the angular information of the principal directions of the lowest rank representation matrix.
3. By combining sgLRR with the Ncuts clustering method, we apply the sgLRR method to multi-cancer sample clustering, and extensive experiments are conducted on gene expression data. Compared with other methods, our methodology has better performance in multi-cancer sample clustering.

The remainder of this paper is outlined as follows: The section 2 summarizes the LRR method, and gives a brief review of some related work. And then, we describe the proposed sgLRR method in detail. In section 3, based on The Cancer Genome Atlas (TCGA) dataset [28], we perform a large number of comparative experiments to demonstrate the sgLRR method with better performance on the multi-cancer sample clustering. And, we discuss and analysis the experimental results from different aspects. In section 4, we describe the corresponding discussion. In the section 5, we summarize our work for the full paper.

## Methods

First, we review the related work about the low-rank representation. And then, then we introduced our proposed approach.

### Related work

In recent years, the LRR method and its improved algorithms have been widely used in many fields. Furthermore, the group theory based on manifold learning has also captured the attention of the researchers. In subsections ***Low-Rank Representation*** and ***The Symmetric Constraint for the Low-Rank Representation***, we review the LRR method [9] and the symmetric constraint for the low-rank representation [16], respectively. Then, in subsection ***Manifold Learning for Graph Regularization***, we give a detail introduction to graph regularization based on manifold learning [29, 30].

#### Low-Rank Representation

Learning a lowest rank representation matrix of the observation dataset is the aim of LRR method [9]. Given an observation data matrix **X** = [**x**_1_, **x**_2_, ..., **x**_*n*_] ∈ *ℝ*^*m* × *n*^ with no error. And, there is an overcomplete dictionary matrix **A** = [**a**_1_, **a**_2_, ..., **a**_*k*_] ∈ *ℝ*^*m* × *k*^ and a union of multiple low-dimensional independent subspaces. It is assumed that the subspaces can be linearly spanned by the dictionary matrix **A**. Therefore, the given observation data **X** can be represented by these low-dimensional subspaces, and the relationship between data **X** and matrix **A** is **X** = **AZ**. In other words, data **X** is a linear combination of the dictionary matrix **A**. The function of the LRR method is as follows:

1 $$ \underset{\mathbf{Z}}{\min}\mathit{\operatorname{rank}}\left(\mathbf{Z}\right)\kern0.75em \mathrm{s}.\mathrm{t}.\mathbf{X}=\mathbf{AZ}. $$

Here, **X** = [**x**_1_, **x**_2_, ..., **x**_*n*_] ∈ *ℝ*^*m* × *n*^ is the observation data matrix. *m* is the total number of features, and *n* is the total number of the samples. **A** = [**a**_1_, **a**_2_, ..., **a**_*k*_] is the overcomplete dictionary matrix, and **Z** = [**z**_1_, **z**_2_, ..., **z**_*n*_] ∈ *ℝ*^*k* × *n*^ is the low rank representation matrix. The element **z**_*i*_ of matrix **Z** is the mapping relationship from {**x**_*i*_| **x**_*i*_ ∈ **X**^*m* × *n*^, 1 ≤ *i* ≤ *n*} to the dictionary matrix **A**. In general, the matrix **Z** is also called the coefficient matrix, and it is a new expression form of **X** that is based on the dictionary matrix **A**. The purpose of the LRR method is to find the lowest rank representation matrix **Z**^∗^.

In practical analysis, the observation data matrix **X** is usually selected as the dictionary matrix **A**, which is a very important aspect of the LRR [9, 15, 26, 27]. According to the matrix multiplication rule, the lowest rank representation matrix **Z** = [**z**_1_, **z**_2_, ..., **z**_*n*_] ∈ *ℝ*^*n* × *n*^ is a square matrix. Equation (1) can be rewritten as follows:

2 $$ \underset{\mathbf{Z}}{\min}\mathit{\operatorname{rank}}\left(\mathbf{Z}\right)\kern0.75em \mathrm{s}.\mathrm{t}.\mathbf{X}=\mathbf{XZ}. $$

In this case, the matrix **Z**^∗^ represents the relation of each sample of **X**. In other words, the element ${\mathbf{z}}_{ij}^{\ast }$ of matrix **Z**^∗^ represents the similarity between the samples **x**_*i*_ and **x**_*j*_. Therefore, the element ${\mathbf{z}}_{ij}^{\ast }$ should be equal to the element ${\mathbf{z}}_{ji}^{\ast }$. That is, the matrix **Z**^∗^ is a symmetric matrix when the observation data matrix **X** is clean.

Because the rank function is nonconvex, no closed expression can be found. Therefore, Eq. (2) is very difficult to solve. The related research has shown that the nuclear-norm of a matrix is a minimal convex envelope of the rank of the matrix [31–33]. Therefore, Eq. (2) is equivalent to the following nuclear-norm convex optimization problem:

3 $$ \underset{\mathbf{Z}}{\min }{\left\Vert \mathbf{Z}\right\Vert}_{\ast}\kern0.75em \mathrm{s}.\mathrm{t}.\mathbf{X}=\mathbf{XZ}. $$

Here, ‖⋅‖_∗_ is the nuclear-norm. It is the sum of all singular values of the matrix **Z**, which is the minimal convex envelope of the rank function [17]. In the actual situation, the observation data are inevitably polluted by noise or outliers under certain special circumstances. Therefore, a certain regularization constraint ‖⋅‖_*l*_ is usually added to (3) to balance the interference. The improved formula is as follows:

4 $$ \underset{\mathbf{Z},\mathbf{E}}{\min }{\left\Vert \mathbf{Z}\right\Vert}_{\ast }+\gamma {\left\Vert \mathbf{E}\right\Vert}_l\kern0.75em \mathrm{s}.\mathrm{t}.\mathbf{X}=\mathbf{XZ}+\mathbf{E}. $$

Here, the matrix **E** denotes the noise or outliers. The parameter *γ* > 0 is to balance the adaptability of each part in (4), and ‖⋅‖_*l*_ is regularization constraint. In general, the appropriate regularization constraint ‖⋅‖_*l*_ is selected according to different types of noise and outliers in real environments. For example, ‖⋅‖_2, 1_, i.e., the *l*_2, 1_ norm, is used to extract the sample-specific corruptions and small noise or outliers, and ‖⋅‖_0_, i.e., the *l*_0_ norm, is used to deal with the significant noise or outliers [27]. Solving the *l*_0_ norm is an NP-hard problem. Therefore, it is usually replaced by ‖⋅‖_1_, i.e., the *l*_1_ norm.

The above is a description of the classic original LRR method. The LRR method deals with the observation data from a holistic perspective. That is, the global structural features of the observation data are represented by the lowest rank representation matrix **Z**^∗^. In addition, the LRR method maps the structures of the observation data from high-dimensional spaces to low-dimensional spaces. It reduces the difficulty of processing high-dimensional observation data.

#### The Symmetric Constraint for the Low-Rank Representation

**Fig. 1:**
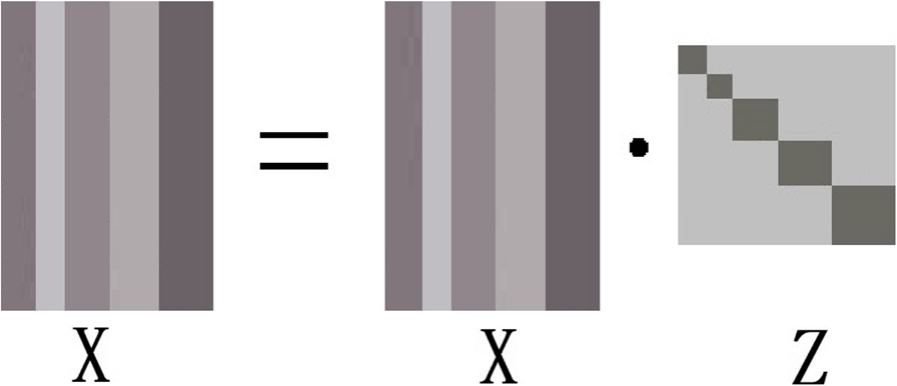
The LRR method with clean data

**Fig. 2:**
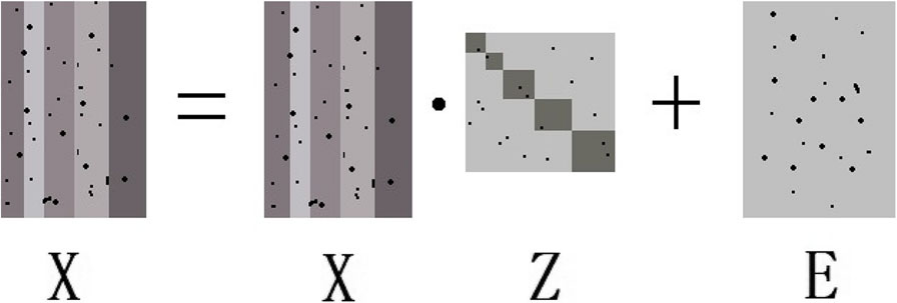
The LRR method with real data

With clean observation data, based on function (3), the most ideal matrix **Z**^∗^ is a block diagonal and strictly symmetric matrix, as shown in Fig. 1. However, according to function (4), the lowest rank representation matrix **Z**^∗^ is not strictly symmetric when using real data with noise and outliers, as shown in Fig. 2 [34].. In other words, because the element **Z**_*ij*_ is not equal to the element **Z**_*ji*_, the degree of similarity of the *i*-th sample to the *j*-th sample is not equal to the degree of similarity of the *i*-th of sample to the *j*-th sample. One question worth considering is which of the two elements is more suitable to be used to reflect the similarity between the two samples.

In general, an affinity matrix is usually constructed using symmetric operation, i.e., (| **Z**^∗^| +|**Z**^∗^|^T^)/2, to reflect the similarity of samples. Then, based on the affinity matrix, spectral clustering algorithms generally use the Ncuts clustering method for subspace clustering. To avoid symmetric operations, Chen et al. imposed a symmetric constraint onto the LRR to obtain a symmetric lowest rank representation matrix [16]. The improved method was named the low-rank representation with symmetric constraint (LRRSC) and it can be expressed as follows:

5 $$ \underset{\mathbf{Z},\mathbf{E}}{\min }{\left\Vert \mathbf{Z}\right\Vert}_{\ast }+\gamma {\left\Vert \mathbf{E}\right\Vert}_l\kern0.75em \mathrm{s}.\mathrm{t}.\mathbf{X}=\mathbf{XZ}+\mathbf{E},\mathbf{Z}={\mathbf{Z}}^{\mathrm{T}}. $$

The symmetric lowest rank representation matrix **Z**^∗^ can greatly preserve the subspace structures of the observation data. Therefore, the affinity matrix based on the principal direction angular information of the symmetric lowest rank representation matrix **Z**^∗^ can effectively reflect the similarity between samples. However, the LRRSC method does not consider the local geometrical structural information. It may lose important information when obtaining the lowest rank representation matrix. In the next section, we use manifold learning with graph regularization to solve this problem.

#### Manifold Learning for Graph Regularization

In actual situations, the given observation data **X** ∈ *ℝ*^*m* × *n*^ are usually high-dimensional. Thus, the local geometrical structural information exists at each data point and at its *k*-nearest-neighboring data points. Capturing the local geometrical structural information is important for the performance of subspace clustering. Fortunately, graph regularization based on manifold learning provides a feasible option to achieve this aim [29]. This approach can preserve the intrinsic local geometrical structures that are embedded in the high-dimensional data space.

In graph theory, the “manifold assumption” is that data points near local geometrical structures should keep their proximity under a new basis [35]. If we map the adjacent data points **x**_*i*_ and **x**_*j*_ in the high-dimensional space to the low-dimensional space, their mapping data points **z**_*i*_ and **z**_*j*_ should be close in the low-dimensional space. Therefore, the local geometrical structural information of the data points **x**_*i*_ and **x**_*j*_ can be represented in the low-dimensional space. In other words, if the characteristics of the data points are similar in the high-dimensional space, their mapping data points can be clustered into the same class in the low-dimensional space.

We take each data point as a vertex. The data points are defined by the column of observation data **X** = [**x**_1_, **x**_2_, ..., **x**_*n*_]. Therefore, the number of vertices is *n*. All *n* vertices form a graph **G**. The weight of the connected edge of vertices *i* and *j* in the graph **G** is represented by **w**_*ij*_. The assignment rule of **w**_*ij*_ is as follows:

6 $$ {\mathbf{w}}_{ij}=\Big\{{\displaystyle \begin{array}{l}1\ \mathrm{if}\ {\mathbf{x}}_i\in {\mathbf{N}}_k\left({\mathbf{x}}_j\right)\ \mathrm{or}\ {\mathbf{x}}_j\in {\mathbf{N}}_k\left({\mathbf{x}}_i\right)\\ {}0\ \mathrm{otherwise}\end{array}}, $$

where **N**_*k*_(**x**_*i*_) denotes the set of the *k*-nearest-neighbors of **x**_*i*_. As suggest in [26, 27], we select the *k* = 5 as the nearest neighbors for the experimental datasets. In addition, all elements **w**_*ij*_ make up a symmetric weight matrix **W**.

In the low-dimensional space, the new relationship of the data points is as follows:

7 $$ \underset{\mathbf{Z}}{\min }{\sum}_{ij}{\left\Vert {\mathbf{z}}_i-{\mathbf{z}}_j\right\Vert}^2{\mathbf{w}}_{ij}. $$

After a linear algebraic transformation, the above optimization problem (7) can be written as follows:

8 $$ \underset{\mathbf{Z}}{\min } tr\left({\mathbf{Z}}^{\mathrm{T}}\mathbf{LZ}\right). $$

Here, *tr*(⋅) is the trace of the matrix. **L** is called the graph Laplacian matrix. It is defined by **L** = **D** − **W**. The matrix **D** is a diagonal matrix, and the element **d**_*ii*_ of **D** is sum of the *i*-th row of **W**, i.e., ${\mathbf{d}}_{ii}={\sum}_j^n{\mathbf{w}}_{ij}$.

### sgLRR methodology

In this section, we introduce our method for multi-cancer sample clustering. First, we obtain the objective function according to the problem’s formulation and solve the function using the linearized adaptive direction method with the adaptive penalty (LADMAP) method [36]. Then, we provide the complete algorithm for easier understanding. Second, we combined our method with the Ncuts clustering method by learning an affinity matrix. Finally, the proposed method is used for the subspace segmentation of multi-cancer sample clustering.

### Problem formulation and the solution

In this subsection, our goal is to propose a novel LRR model to preserve the intrinsic local geometrical structures of the observation data and simultaneously weaken the effects of noise and outliers in the dictionary matrix. We introduce graph regularization based on manifold learning and the symmetric constraint into the original LRR method. It is as follows:

9 $$ {\displaystyle \begin{array}{l}\underset{\mathbf{Z},\mathbf{E}}{\min }{\left\Vert \mathbf{Z}\right\Vert}_{\ast }+\beta tr\left(\mathbf{ZL}{\mathbf{Z}}^{\mathrm{T}}\right)+\gamma \left\Vert \mathbf{E}\right\Vert {}_1\kern0.75em \\ {}\mathrm{s}.\mathrm{t}.\mathbf{X}=\mathbf{XZ}+\mathbf{E},\mathbf{Z}={\mathbf{Z}}^{\mathrm{T}},\end{array}} $$

where *β* and *γ* are penalty parameters, **L** is the Laplacian matrix, and ‖ ⋅ ‖_1_ is the regularization constraint. ‖**E**‖_1_ is the sum of the absolute values of each element in the matrix **E**.

In addition, according to the sparsity-based clustering method, e.g., sparse coding combined with clustering, the sparsity constraint can be thought of as a strategy for information localization [37]. Thus, the coefficient matrix with the sparsity constraint can improve the performance of subspace clustering. Namely, by combining the low-rank and sparse data, the within-class affinities are dense, and the between-class affinities are zeros. So, we introduce the sparsity constraint into Eq. (9), and the finally objective function of our method is as follows:

10 $$ {\displaystyle \begin{array}{l}\underset{\mathbf{Z},\mathbf{E}}{\min }{\left\Vert \mathbf{Z}\right\Vert}_{\ast }+\lambda \left\Vert \mathbf{Z}\right\Vert {}_1+\beta tr\left(\mathbf{ZL}{\mathbf{Z}}^{\mathrm{T}}\right)+\gamma \left\Vert \mathbf{E}\right\Vert {}_1\ \\ {}\kern0.5em \mathrm{s}.\mathrm{t}.\mathbf{X}=\mathbf{XZ}+\mathbf{E},\mathbf{Z}={\mathbf{Z}}^{\mathrm{T}},\end{array}} $$

where *λ* is the penalty parameter, and ‖**Z**‖_1_ is the sparsity constraint for the low-rank representation matrix **Z**.

We call the objective function in (10) the graph regularized low-rank representation under combined the sparse and symmetric constraints (sgLRR) method. To obtain a globally optimal solution, we adopt the LADMAP to solve problem (10).

First, we introduce the auxiliary variable **J** to separate variables. It is as follows:

11 $$ {\displaystyle \begin{array}{l}\underset{\mathbf{Z},\mathbf{E}}{\min }{\left\Vert \mathbf{Z}\right\Vert}_{\ast }+\lambda \left\Vert \mathbf{J}\right\Vert {}_1+\beta tr\left(\mathbf{ZL}{\mathbf{Z}}^{\mathrm{T}}\right)+\gamma \left\Vert \mathbf{E}\right\Vert {}_1\kern0.50em \\ {}\ \mathrm{s}.\mathrm{t}.\mathbf{X}=\mathbf{XZ}+\mathbf{E},\mathbf{Z}={\mathbf{Z}}^{\mathrm{T}},\mathbf{Z}=\mathbf{J}.\end{array}} $$

Second, problem (11) can be converted into an unconstrained optimization problem by using the augmented Lagrange multiplier method (ALM) [38]. The formula is rewritten as follows:

12 $$ {\displaystyle \begin{array}{l}\ell \left(\mathbf{Z},\mathbf{E},\mathbf{J},{\mathbf{Y}}_1,{\mathbf{Y}}_2\right)={\left\Vert \mathbf{Z}\right\Vert}_{\ast }+\lambda \left\Vert \mathbf{J}\right\Vert {}_1+\beta tr\left(\mathbf{Z}\mathbf{L}{\mathbf{Z}}^{\mathrm{T}}\right)+\gamma \left\Vert \mathbf{E}\right\Vert {}_1\\ {}\kern7.25em +\left\langle {\mathbf{Y}}_1,\mathbf{X}-\mathbf{XZ}-\mathbf{E}\right\rangle +\left\langle {\mathbf{Y}}_2,\mathbf{Z}-\mathbf{J}\right\rangle \\ {}\kern7.5em +\frac{\mu }{2}\left({\left\Vert \mathbf{X}-\mathbf{XZ}-\mathbf{E}\right\Vert}_F^2+{\left\Vert \mathbf{Z}-\mathbf{J}\right\Vert}_F^2\right),\end{array}} $$

Here, ‖⋅‖_*F*_ is the Frobenius-norm; *λ*, *β*, *λ* and *μ* are the penalty parameters; 〈**A**, **B**〉 = *tr*(**A**^*T*^**B**) represents the Euclidean inner product between the two matrices, and **Y**_1_ and **Y**_2_ are Lagrangian multipliers.

According to the LADMAP method, problem (12) is divided into three problems. They are as follows:

13 $$ {\displaystyle \begin{array}{l}{\ell}_1\left(\mathbf{Z}\right)={\left\Vert \mathbf{Z}\right\Vert}_{\ast }+\beta tr\left(\mathbf{Z}\mathbf{L}{\mathbf{Z}}^{\mathrm{T}}\right)+\left\langle {\mathbf{Y}}_1,\mathbf{X}-\mathbf{XZ}-\mathbf{E}\right\rangle \\ {}\kern2.25em +\left\langle {\mathbf{Y}}_2,\mathbf{Z}-\mathbf{J}\right\rangle +\frac{\mu }{2}\left({\left\Vert \mathbf{X}-\mathbf{XZ}-\mathbf{E}\right\Vert}_F^2+{\left\Vert \mathbf{Z}-\mathbf{J}\right\Vert}_F^2\right),\end{array}} $$

14 $$ {\ell}_2\left(\mathbf{E}\right)=\gamma \left\Vert \mathbf{E}\right\Vert {}_1+\left\langle {\mathbf{Y}}_1,\mathbf{X}-\mathbf{XZ}-\mathbf{E}\right\rangle +\frac{\mu }{2}{\left\Vert \mathbf{X}-\mathbf{XZ}-\mathbf{E}\right\Vert}_F^2, $$

15 $$ {\ell}_3\left(\mathbf{J}\right)=\lambda \left\Vert \mathbf{J}\right\Vert {}_1+\left\langle {\mathbf{Y}}_2,\mathbf{Z}-\mathbf{J}\right\rangle +\frac{\mu }{2}{\left\Vert \mathbf{Z}-\mathbf{J}\right\Vert}_F^2. $$

Problem (13) can be replaced by solving the following problem (16):

16 $$ \min {\left\Vert \mathbf{Z}\right\Vert}_{\ast }+\left\langle {\nabla}_{\mathbf{Z}}\mathbf{q}\left({\mathbf{Z}}_k\right),\mathbf{Z}-{\mathbf{Z}}_k\right\rangle +\frac{\eta_1}{2}{\left\Vert \mathbf{Z}-{\mathbf{Z}}_k\right\Vert}_F^2, $$

where $$ {\nabla}_{\mathbf{Z}}\mathbf{q}\left({\mathbf{Z}}_k\right)=\beta \left({\mathbf{Z}}_k{\mathbf{L}}^{\mathrm{T}}+{\mathbf{Z}}_k\mathbf{L}\right)+{\mu}_k\left({\mathbf{Z}}_k-{\mathbf{J}}_k+{\mathbf{Y}}_2^k/{\mu}_k\right)+{\mu}_k{\mathbf{X}}^{\mathrm{T}}\left(\mathbf{X}{\mathbf{Z}}_k-\mathbf{X}+{\mathbf{E}}_k-{\mathbf{Y}}_1^k/{\mu}_k\right) $$, $$ {\eta}_1=2\beta {\left\Vert \mathbf{L}\right\Vert}_2+{\mu}_k\left(1+{\left\Vert \mathbf{X}\right\Vert}_2^2\right) $$.

We use the following *Lemma-1* to solve problem (16). Chen et al. have given the rigorous mathematical derivations and detailed proofs for this theorem [16].

#### Lemma 1

Given a square matrix **Q** ∈ *ℝ*^*n* × *n*^, there is a unique closed form solution to solve optimization problem (17).

17 $$ {\mathbf{P}}^{\ast }=\arg \underset{\mathbf{p}}{\min}\frac{1}{\mu }{\left\Vert \mathbf{P}\right\Vert}_{\ast }+\frac{1}{2}{\left\Vert \mathbf{P}-\mathbf{Q}\right\Vert}_F^2\kern0.75em \mathrm{s}.\mathrm{t}.\mathbf{P}={\mathbf{P}}^{\mathrm{T}}. $$

It is as follows:

18 $$ {\mathbf{P}}^{\ast }={\mathbf{U}}_r\left({\boldsymbol{\Sigma}}_r-\frac{1}{\mu}\cdot {\mathbf{I}}_r\right){\mathbf{V}}_r^{\mathrm{T}}. $$

Here, **Σ**_*r*_, **U**_*r*_ and **V**_*r*_ are obtained using $\overset{\sim }{\mathbf{Q}}={\mathbf{U}}_r{\boldsymbol{\Sigma}}_r{\mathbf{V}}_r^{\mathrm{T}}$, which is the skinny SVD of the symmetric matrix $\overset{\sim }{\mathbf{Q}}$. In addition, **Σ**_*r*_ =  *diag* (*σ*_1_, *σ*_2_, ..., *σ*_*r*_) with $\left\{r:{\sigma}_r>\frac{1}{\mu}\right\}$ are the positive singular values of matrix $\overset{\sim }{\mathbf{Q}}$. **U**_*r*_ and **V**_*r*_ are the singular vectors of matrix $\overset{\sim }{\mathbf{Q}}$. Matrix $\overset{\sim }{\mathbf{Q}}$ is obtained by $\overset{\sim }{\mathbf{Q}}=\left(\mathbf{Q}+{\mathbf{Q}}^{\mathrm{T}}\right)/2$ and the skinny SVD only keeps the positive singular values of the normal SVD. **I**_*r*_ is an identity matrix.

According to *Lemma-1*, we set **Q** = **Z**_*k*_ − ∇_**Z**_**q**(**Z**_*k*_)/*η*_1_. Then, we solve problem (16) by using the iterative formula (19):

19 $$ {\mathbf{Z}}_{k+1}^{\ast }={\Theta}_{\frac{1}{\eta_1}}\left(\frac{\mathbf{Q}+{\mathbf{Q}}^{\mathrm{T}}}{2}\right). $$

Here, ${\Theta}_{\varepsilon}\left(\mathbf{A}\right)={\mathbf{U}}_r{\mathbf{S}}_{\varepsilon}\left({\boldsymbol{\Sigma}}_r-\frac{1}{\mu_k}\cdot {\mathbf{I}}_r\right){\mathbf{V}}_r^{\mathrm{T}}$ and **S**_*ε*_(*x*) = sgn(*x*) max(| *x*| −*ε*, 0).

We update **Ε** and **J** by minimizing *ℓ*_2_(**E**) and *ℓ*_3_(**J**). And, **Ε** and **J** are independent of each other in this minimization problem. And then, based on a singular value thresholding algorithm, we obtain the iterative formulas of **Ε** and **J**. We set $\frac{\partial {\ell}_2}{\partial \mathbf{E}}=0$ and $\frac{\partial {\ell}_3}{\partial \mathbf{J}}=0$, respectively. Then, we obtain the following equations.

20 $$ \frac{\partial {\ell}_2}{\partial {\mathbf{E}}_k}={\mu}_k\left({\mathbf{E}}_k-\mathbf{X}+\mathbf{X}{\mathbf{Z}}_{k+1}-{\mathbf{Y}}_1^k/{\mu}_k\right)=0\Rightarrow {\mathbf{E}}_k=\mathbf{X}-\mathbf{X}{\mathbf{Z}}_{k+1}+{\mathbf{Y}}_1^k/{\mu}_k, $$

21 $$ \frac{\partial {\ell}_3}{\partial {\mathbf{J}}_k}={\mu}_k\left[{\mathbf{J}}_k-\left({\mathbf{Z}}_{k+1}+{\mathbf{Y}}_2^k/{\mu}_k\right)\right]=0\kern0.5em \Rightarrow {\mathbf{J}}_k={\mathbf{Z}}_{k+1}+{\mathbf{Y}}_2^k/{\mu}_k. $$

According to the NSHLRR method [24] and the singular value thresholding algorithm [39], the iterative formulas of **E** and **J** are as follows:

22 $$ {\mathbf{E}}_{k+1}=\Psi {}_{\frac{\gamma }{\mu_k}}\left(\mathbf{X}-\mathbf{X}{\mathbf{Z}}_{k+1}+{\mathbf{Y}}_1^k/{\mu}_k\right), $$

23 $$ {\mathbf{J}}_{k+1}=\max \left\{{\Psi}_{\frac{\lambda }{\mu_k}}\left({\mathbf{Z}}_{k+1}+\frac{1}{\mu_k}{\mathbf{Y}}_2^k\right),0\right\}. $$

Based on the above, we discuss the time complexity of sgLRR compared to the original LRR. As described in [36], the complexity of LADMAP method for LRR is *O*(*rmn*), where *r* is the rank of the matrix **Z**, *m* and *n* is the size of observation data matrix **X** ∈ *ℝ*^*m* × *n*^. For sgLRR method, the construction of the k-nearest neighbor graph is *O*(*n*^2^*m*). Therefore, the complexity of sgLRR is *O*(*rmn* + *n*^2^*m*).

Finally, **Algorithm 1** provides the complete sgLRR algorithm.

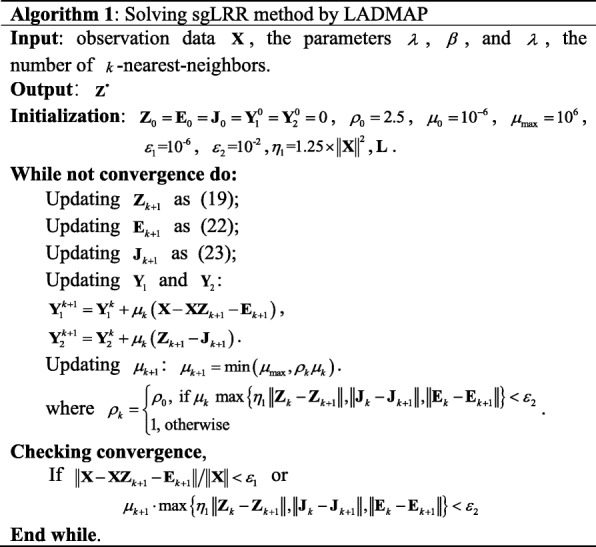



### sgLRR method combined with the Ncuts clustering method

We obtain the lowest rank representation matrix **Z**^∗^ by **Algorithm 1**. The obtained matrix **Z**^∗^ inherits and improves the grouping effect of the LRR method. The symmetric property of matrix **Z**^∗^ strictly reflects the similarity of the data samples, and the data samples that belong to the same group highlight the same subspace of matrix **Z**^∗^. However, as mentioned in [9], the complex application may fail in the lowest rank representation matrix **Z**^∗^ and in fully using the information within matrix **Z**^∗^. Therefore, we combine the sgLRR method with the Ncuts clustering method to guarantee correct segmentation results.

First, we learn an affinity matrix **H** that is the link between the sgLRR method and the Ncuts clustering method. The affinity matrix **H** utilizes the angular similarity information of the principal direction of matrix **Z**^∗^, and matrix **H** is a similar undirected graph that further improves the grouping effect. The process below can be defined as learning matrix **H**.

1. The matrix **Z**^∗^ is decomposed into **Z**^∗^ = **U**^∗^**Σ**^∗^(**V**^∗^)^T^ using skinny SVD.
2. Define the matrix **M** = **U**^∗^(**Σ**^∗^)^1/2^ or the matrix **N** = (**Σ**^∗^)^1/2^(**V**^∗^)^T^. Because the matrix **Z**^∗^ is a symmetrical matrix, both matrix **M** and matrix **N** are equivalent for leaning the affinity matrix **H**.
3. The element of the affinity matrix **H** is calculated using function (24).

24 $$ {\mathbf{H}}_{ij}={\left(\frac{{\mathbf{m}}_i^{\mathrm{T}}{\mathbf{m}}_j}{{\left\Vert {\mathbf{m}}_i\right\Vert}_2{\left\Vert {\mathbf{m}}_j\right\Vert}_2}\right)}^2\kern0.75em \mathrm{or}\kern1em {\mathbf{H}}_{ij}={\left(\frac{{\mathbf{n}}_i^{\mathrm{T}}{\mathbf{n}}_j}{{\left\Vert {\mathbf{n}}_i\right\Vert}_2{\left\Vert {\mathbf{n}}_j\right\Vert}_2}\right)}^2, $$

where **m**_*i*_ is the *i*-th row of **M**, and **n**_*i*_ is the *i*-th row of **N**.

Next, we adopt the Ncuts clustering method to produce the final data sample clustering results. The Ncuts clustering method was proposed by Shi et al. and is closely related to graph theory [40]. This approach can well reflect the degree of similarity within classes and the degree of dissimilarity between classes. This approach has been successfully applied in image segmentation and has numerous successful examples in different fields and datasets, such as gene expression overlapping clustering based on the penalized weighted normalized cut [41].

Finally, we briefly summarize the process of the multi-cancer sample clustering algorithm. It is as follows.

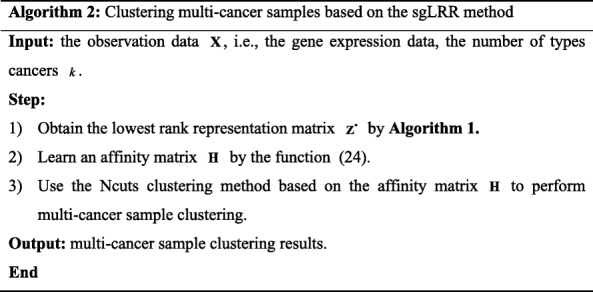



## Results

### Datasets

As the biggest cancer genomic profile database, The Cancer Genome Atlas (TCGA) provides publicly available datasets with over 30 types of cancers using high-throughput sequencing technology and integrated multidimensional analyses to help improve the diagnosis, prevention, and treatment of cancer [28].

We use five real gene expression datasets that were downloaded from the TCGA to construct the integrated datasets for the experiments. The five original datasets are the cholangiocarcinoma (CHOL) dataset, the head and neck squamous cell carcinoma (HNSC) dataset, the colon adenocarcinoma (COAD) dataset, the esophageal carcinoma (ESCA) dataset and the pancreatic adenocarcinoma (PAAD) dataset. Each dataset consists a different number of cancer samples and normal samples, and each sample contains 20,502 genes. Table 1 lists the distribution number of the samples for each dataset.

**Table 1:** The distribution of the samples in the five datasets

Gene Expression Datasets	The Distribution of the Samples in the Datasets		
	Cancer Samples	Normal Samples	Total of Number
COAD	262	19	281
ESCA	183	9	192
CHOL	36	9	45
PAAD	176	4	180
HNSC	398	20	418

Note: The Gene Expression Datasets represent the different cancer sample data: *COAD* colon adenocarcinoma, *ESCA* esophagus cancer, *CHOL* cholangiocarcinoma, *PAAD* pancreatic adenocarcinoma, *HNSC* head and neck squamous cell carcinoma

As listed in the Table 1, we use all the cancer samples of each dataset to construct six integrated datasets. And, the six integrated datasets are named the CO-CH (COAD-CHOL) dataset, the PA-ES (PAAD-ESCA) dataset, the CH-HN-CO (CHOL-HNSC-COAD) dataset, the ES-CH-HN (ESCA-CHOL-HNSC) dataset, the ES-CO-PA-HN (ESCA-COAD-PAAD-HNSC) dataset and the CO-CH-ES-HN (COAD-CHOL-ESCA-HNSC) dataset, respectively. The characteristics of the datasets are as follows: the CO-CH dataset contain all 298 cancer samples from COAD and CHOL; the PA-ES dataset contain all 359 cancer samples from PAAD and ESCA; the CH-HN-CO dataset contain all 696 cancer samples from CHOL, HNSC and COAD; the ES-CH-HN dataset contain all 617 cancer samples ESCA, CHOL and HNSC; the CO-CH-ES-HN dataset contain all 879 cancer samples from COAD, CHOL, ESCA and HNSC; and, the ES-CO-PA-HN dataset contain all 1019 cancer samples from ESCA, COAD, PAAD and HNSC. The distribution of the six datasets are summarized and listed in Table 2. We conduct experiments on the basis of the six datasets to prove the performance of sgLRR method.

**Table 2:** The distribution of the six datasets

Datasets	The number of samples of each type of cancer	Total number of samples	subspace number
CO-CH	262–36	298	2
PA-ES	176–183	359	2
CH-HN-CO	36–398-262	696	3
ES-CH-HN	183–36-398	617	3
CO-CH-ES-HN	262–36–183-398	879	4
ES-CO-PA-HN	183–262–176-398	1019	4

Note: The datasets represent different integrated datasets. The characteristics of each dataset are described in the previous passage

### Measurement metrics for the experiment results

In this article, we use multiple measures to strictly analyze the clustering results. The clustering results are mainly evaluated by the Accuracy (Acc) [42], Matthews Correlation Coefficient (MCC) [43], Rand Index (RI) [44] and Normalized Mutual Information (NMI) [45]. Next, we concisely introduce them.

### Accuracy

The Accuracy (Acc) evaluates the clustering results on the global level by calculating the matching degree of experimental result labels and actual labels. The values of the Acc ranges from 0 to 1, and the higher value is, the better the clustering results is. The specific formula is as follows.

25 $$ Acc=\frac{\sum_{i=1}^n\delta \left({p}_i, map\left({q}_i\right)\right)}{n}\times 100\%. $$

Here, *δ*(*p*_*i*_, *map*(*q*_*i*_)) is defined as follows:

26 $$ \delta \left({p}_i, map\left({q}_i\right)\right)=\Big\{{\displaystyle \begin{array}{l}1,\mathrm{if}\ {p}_i= map\left({q}_i\right)\\ {}0,\mathrm{otherwise}\end{array}}, $$

where *n* is the number of data samples, *p*_*i*_ is the real label for the *i*-th sample, and *q*_*i*_ is the experimental result of the *i*-th sample. *map*(*q*_*i*_) is the mapping function that can match the clustering result to the real label using the Kuhn-Munkres approach [46].

### Matthews correlation coefficient

**Fig. 3:**
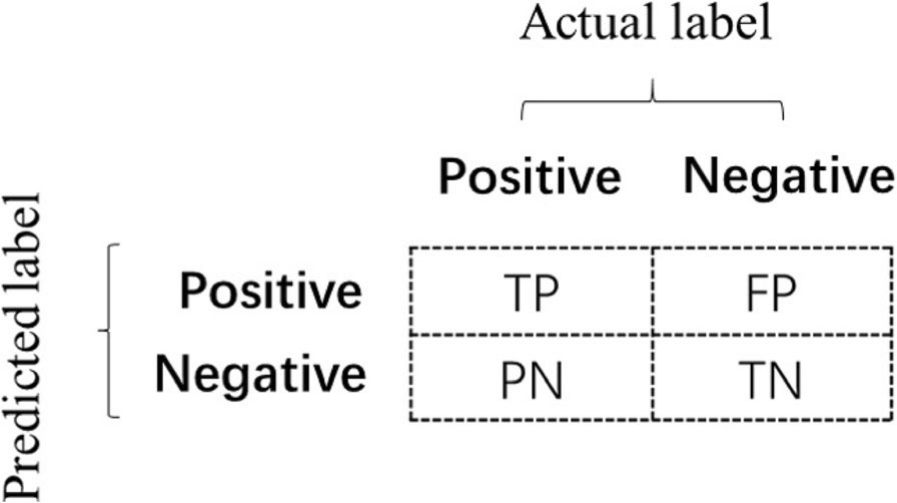
The item of *TP*, *FP*, *TN*, *FN*

The Matthews Correlation Coefficient (MCC) is widely used performance measure in biomedical research to handle imbalanced datasets [43, 47–49]. In general, MCC represents a comprehensive evaluation measure which has a better balance of both aspects of the accuracy and coverage than the individual precision and recall values [49]. The MCC is defined in terms of *TP* (True Positive), *FP* (False Positive) and *TN* (True Negative), *FN* (False Negative), and its formula is as follows.

27 $$ MCC=\frac{TP\times TN- FP\times FN}{\sqrt{\left( TP+ FP\right)\left( TP+ FN\right)\left( TN+ FP\right)\left( TN+ FN\right)}}\times 100\%, $$

where *TP* is the number of true positives, where the data points that actually belong to the same cluster are grouped into the same cluster in the experiment results. *TN* is the number of true negatives, where the data points that actually belong to the same cluster are grouped into the different clusters in the experiment results. *FP* is the number of false positives, where the data points that actually belong to different clusters are grouped into the same cluster in the experiment results. *FN* is the number of false negatives, where the data points that actually belong to different clusters are grouped into the different clusters in the experiment results. The Fig. 3 shows *TP*, *TN*, *FP*, *FN* clearly.

**Fig. 4:**
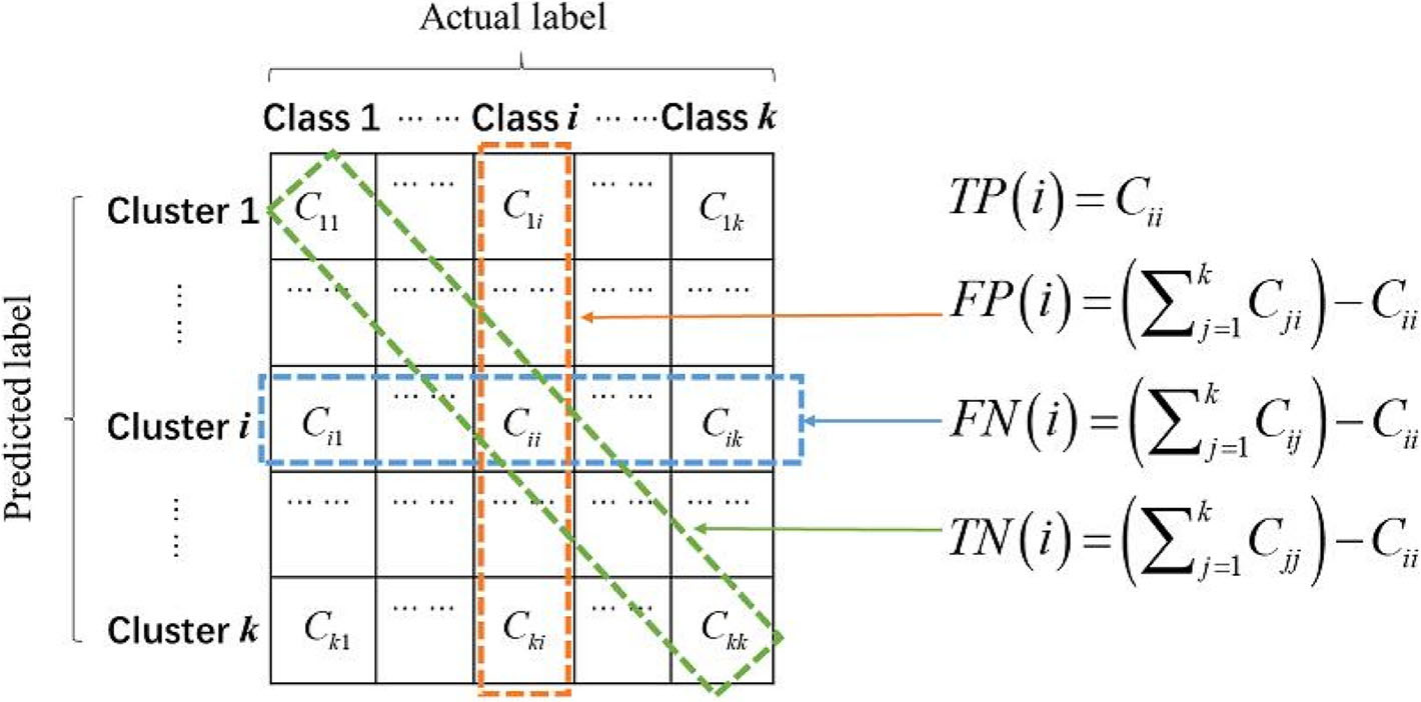
Confusion matrix and the item of *TP*, *FP*, *TN*, *FN* for multi-cancer dataset clustering

The value of MCC is took in the interval [−1, 1], with 1 representing a complete agreement, −1 indicating a complete disagreement, and 0 indicating that the clustered result was uncorrelated with the ground truth [50]. For the multi-class dataset clustering with *k* classes, the MCC can be calculated by the confusion matrix. And, the confusion matrix is a matrix **C** = (*C*_*ij*_)_*k* × *k*_ with the size of *k* × *k*, where *C*_*ij*_ represents the number of samples, which in actually belongs to the class *i*, are clustered to be in the cluster *j*. And, the confusion matrix and the item of *TP, FP, TN, FN* for multi-cancer samples clustering are defined as the Fig. 4.

### Rand index

The Rand Index (RI) is an objective criterion for the evaluation of clustering methods. From a mathematical standpoint, the RI is related to the Acc, but it is applicable even when class labels are not used [44]. Given the set of *n* data points *S* = {*O*_1_, *O*_2_, ..., *O*_*n*_} that are to be clustered, the specific partitions *V* = {*v*_1_, *v*_2_, ..., *v*_*r*_} and *U* = {*u*_1_, *u*_2_, ..., *u*_*c*_} are the clustering results of *S* that are divided into *r* and *c* disjointed sets, respectively. If *V* represents the true results and *U* represents the experiment results, then RI is defined as follows.

28 $$ RI=\frac{a+d}{a+b+c}\times 100\%, $$

$$ \mathrm{where}\Big\{{\displaystyle \begin{array}{l}a\ \mathrm{is}\ \mathrm{the}\ \mathrm{total}\ \mathrm{of}\ \mathrm{the}\ \mathrm{data}\ \mathrm{pairs}\ \mathrm{that}\ \mathrm{exist}\ \mathrm{in}\ \mathrm{the}\ \mathrm{same}\ \mathrm{cluster}\ \mathrm{for}\ V\ \mathrm{and}\ U.\\ {}b\ \mathrm{is}\ \mathrm{the}\ \mathrm{total}\ \mathrm{of}\ \mathrm{the}\ \mathrm{data}\ \mathrm{pairs}\ \mathrm{that}\ \mathrm{exist}\ \mathrm{in}\ \mathrm{the}\ \mathrm{different}\ \mathrm{cluster}\mathrm{s}\ \mathrm{both}\ \mathrm{for}\ V\ \mathrm{and}\ U.\\ {}c\ \mathrm{is}\ \mathrm{the}\ \mathrm{case}\ \mathrm{with}\ \mathrm{different}\kern0.5em \mathrm{the}\ a\ \mathrm{and}\ b.\end{array}} $$

The value of the RI ranges from 0 to 1, and the higher value is, the better the clustering results is.

### Normalized mutual information

The Normalized Mutual Information (NMI) is commonly used in clustering to measure the similarity of two clustering results [45]. There are the clusters *Ξ* = [*ξ*_*i*_]_*k*_ obtained by the clustering algorithm and the true inherent classes *Ω* = [*ω*_*i*_]_*k*_. The NMI is defined as follows.

29 $$ NMI\left(\Xi, \Omega \right)=2\times \frac{I\left(\Xi; \Omega \right)}{\left(H\left(\Xi \right)+H\left(\Omega \right)\right)}\times 100\%. $$

Here, $I\left(\Xi; \Omega \right)=\sum \limits_{\xi_i\in \Xi}^k\sum \limits_{\omega_i\in \Omega}^kp\left(\xi, \omega \right)\log \left(\frac{p\left(\xi, \omega \right)}{p\left(\xi \right)p\left(\omega \right)}\right)$ is the mutual information, and $H\left(\Xi \right)=\sum \limits_{i=1}^kp\left({\xi}_i\right)\times I\left({\xi}_i\right)=\sum \limits_{i=1}^kp\left({\xi}_i\right)\times {\log}_2\left(\frac{1}{p\left({\xi}_i\right)}\right)$, where *p*(*ξ*_*i*_) (*p*(*ω*_*i*_)) is the probability of an object being in *cluster ξ*_*k*_ (class *ω*_*k*_), and *p*(*ξ*_*k*_, *ω*_*j*_) is the joint probability that an object lies in cluster *ξ*_*k*_ and class *ω*_*k*_. The value of the NMI ranges from 0 to 1, and the higher value is, the better the clustering results is.

### Experiment result and discussion

In this subsection, we cluster the multi-cancer samples using the sgLRR method and compare the results with other related methods to analyze the performance. The related methods in the comparative experiments include K-means, T-SNE, LLE, NMF [42], PCA [33], LRR [9], LLRR [26] and MLLRR [27]. And, the experiments are carried out on the six integrated datasets. We obtain the clustering results of the sgLRR method and the comparison methods. For the compared methods: K-means, T-SNE, LLE, NMF, PCA, LRR, LLRR, and MLLRR, they are the traditional existing clustering and dimensional reduction methods. And, we categorize these methods into three classes. The first kind of method is the classic method for clustering, including K-means, NMF and PCA. The T-SNE and LLE belong to the second kind of method for dimensional reduction. T-SNE and LLE both are well-established manifold learning methods. Among them, T-SNE is almost the dominant one in bioinformatics, especially for expression data. The third kind of method include LRR, LLRR, MLRR and our proposed sgLRR method. These methods belong to the subspaces clustering methods to dimensional reduction by low rank representation the raw datasets.

In addition, K-means clustering algorithm is usually used to obtain the final clustering result in spectral clustering. In this paper, K-means method uses the K-means + + algorithm for cluster center initialization and squared Euclidean distance by default, and in K-means + + algorithm, the initial cluster center is randomly selected [51]. Therefore, if K-means method is used to repeat the experiment with the same dataset, the results of these experiments will not be identical, and there will be minor differences. This difference will affect our performance evaluation of clustering methods. In our experiments, in order to improve the reasonable of results and reduce the difference, we repeat clustering experiment 50 times. And, the mean of results is taken as the measurements of clustering results.

The experimental results are listed in the Table 3. And, we highlight the best clustering results in bold. Of all the best results, except for the few results, the results obtained by sgLRR method are overwhelmingly superior in the nine experimental methods. In the next, based on the clustering results, we detailed discuss and analyze the advantage of sgLRR method which is different with the above comparison methods. And, the details are as follows.

**Fig. 5:**
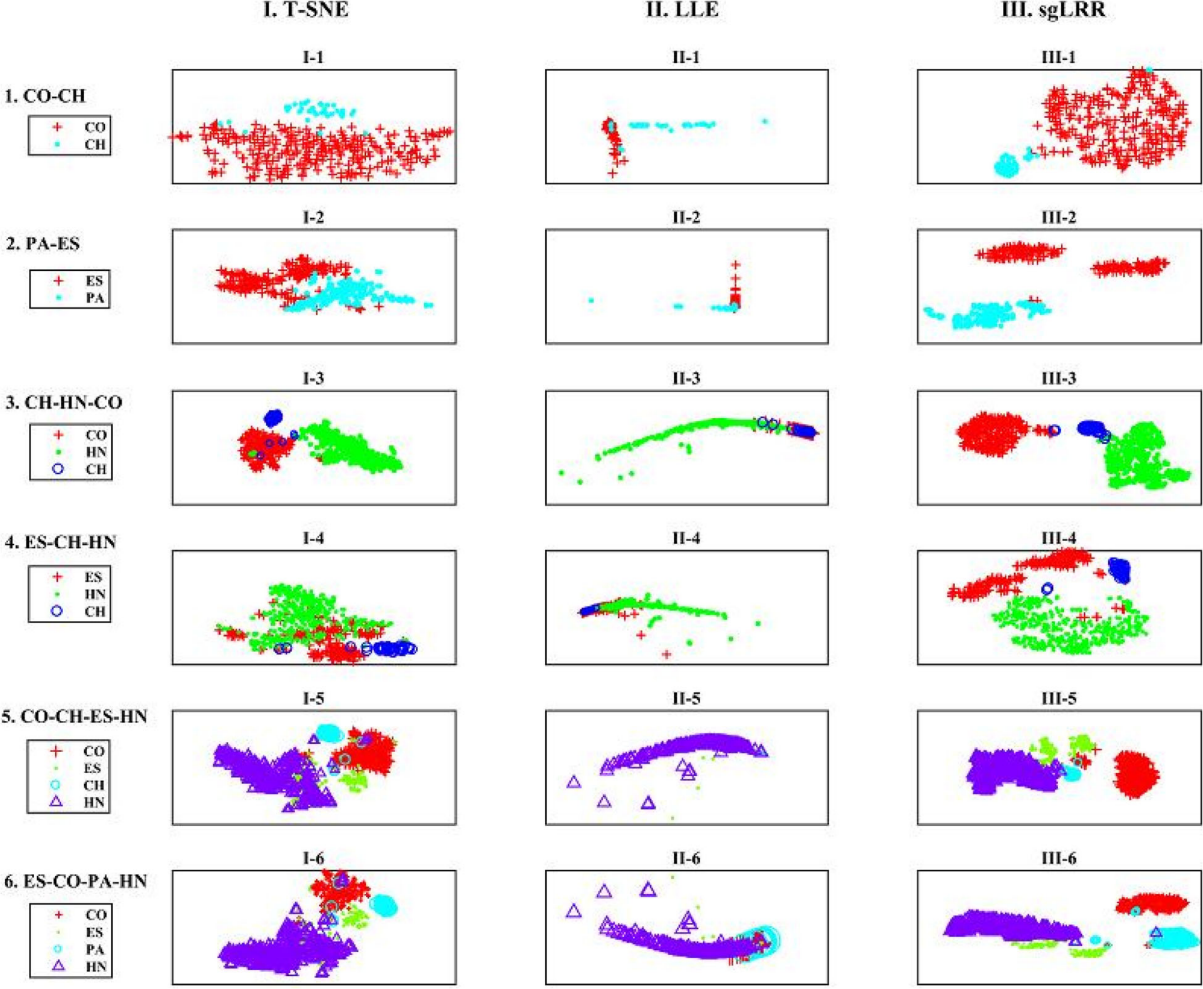
Visualization of the dimensionality reduction data of obtained by T-SNE, LLE and sgLRR methods for the six integrated datasets

**Fig. 6:**
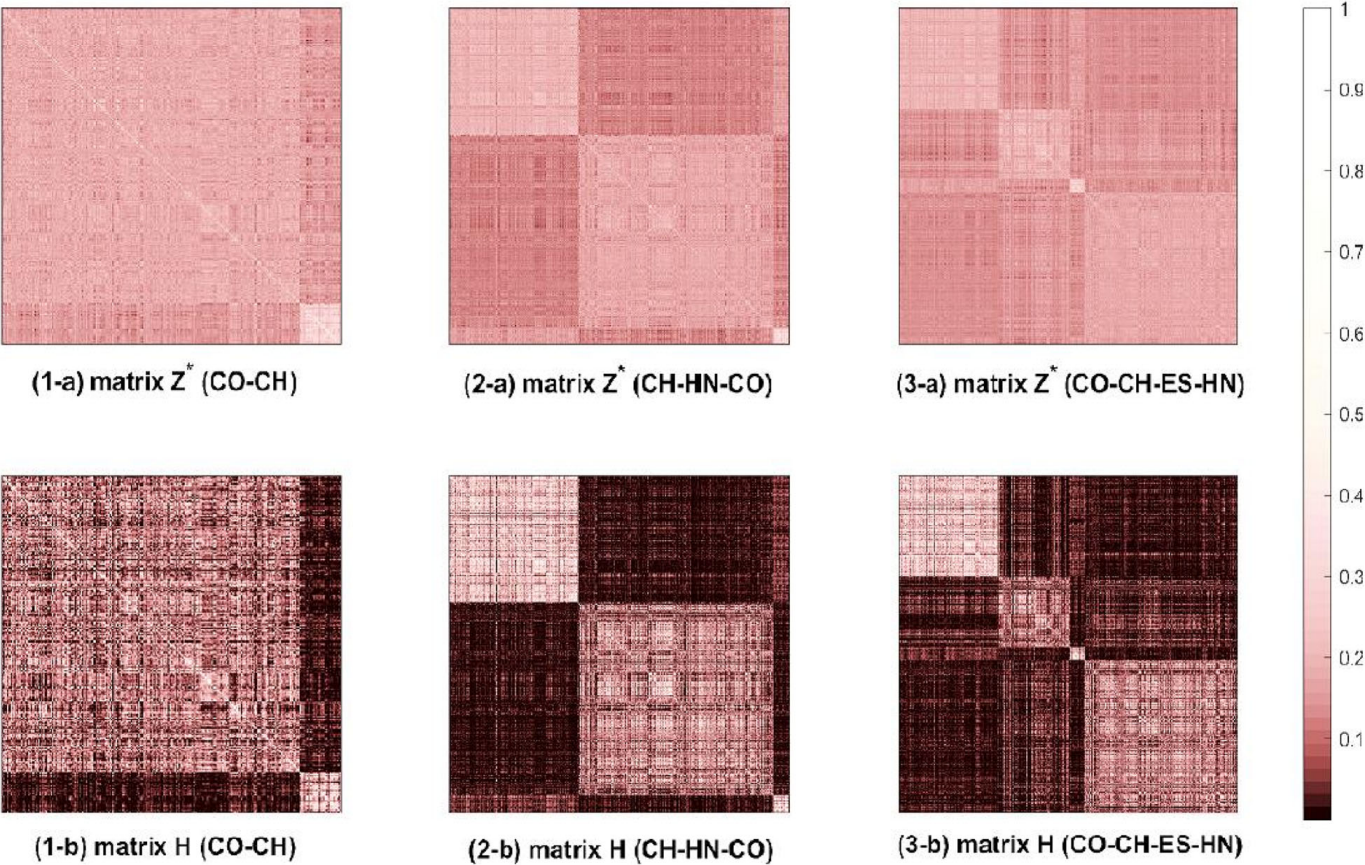
The heat maps intuitively compare the grouping effects of matrices **Z**^∗^ and **H**. (*1-a*) and (*1-b*) are the heat maps based on the matrices **Z**^∗^ and **H** for the CO-CH dataset, respectively; (*2-a*) and (*2-b*) are the heat maps based on the matrices **Z**^∗^ and **H** for the CH-HN-CO dataset, respectively; and (*3-a*) and (*3-b*) are the heat maps based on the matrices **Z**^∗^ and **H** for the CO-CH-ES-HN dataset, respectively

1. For the most of metrics results in the Table 3, the LRR, LLRR, MLRR and sgLRR methods are better than the first kind method, including K-means, NMF and PCA. Furthermore, the performances of LLRR, MLRR and sgLRR methods improve as the number of cancer types increasing. Notably, the best clustering results are mainly obtained by the sgLRR method. From an overall standpoint, the experimental results demonstrate that the methods for the low-rank representation are better for multiple subspace clustering than the classical clustering method. One mainly reason is that the low-rank representation methods with the characteristics of capturing the subspace structure of datasets. Therefore, the gene expression data structures of each type cancer are stored in their respective low dimensional subspaces, which makes the different types of cancer dataset more separable.
2. In order to demonstrate dimensionality reduction datasets of sgLRR with the better performance on the multi-cancer gene expression datasets, we compare sgLRR method with the second class of method: T-SNE and LLE. At first, we visualize the dimensionality reduction datasets which are obtained by the T-SNE, LLE and sgLRR methods, as shown in Fig. 5. And, the data points are colored according to their actual labels. As shown in the Fig. 5, in the dimensionality reduction data obtained by the T-SNE method, there are several overlaps between clusters of different types of cancer samples such as I-2, I-4, I-5 and I-6. In the dimensionality reduction data obtained by the LLE method, the separability of clusters of different types of cancers is not obvious such as II-3, II-5 and II-6. In the dimensionality reduction data obtained by the sgLRR method, the independence of different cancer sample clusters is obvious such as III-2, III-4 and III-6, and the data subspace has better separability effect than T-SNE and LLE methods. Therefore, we come to the conclusion that the separability among different types cancers in the dimensionality reduction data of three methods is best by sgLRR method, followed by T-SNE method, and finally by LEE method. Moreover, sgLRR method makes the data points more separable between classes than T-SNE method. That is due to that sgLRR method combines the low-rank representation method with the graph regularization constraint based on manifold learning, which enhances the separable between different types of cancer data in dimensionality reduction datasets.
3. In the third kind of method, what LRR LLRR MLLRR and sgLRR methods have in common is that they represent global structure of the raw dataset by a low-rank matrix with low dimensional subspaces. However, comparing the LRR method with the LLRR, MLLRR and sgLRR methods, the clustering results of most datasets are better than the LRR method. This is because LLRR, MLLRR and sgLRR methods with graph regularization based on manifold learning can capture the inherent geometric structural information of datasets. The results suggest that introducing graph regularization based on manifold learning can improve the clustering performance of the method. Moreover, we can find that the most of metrics of the sgLRR are higher than those of the LLRR, and they are also the best in all comparison methods. This is because the symmetry constraint weakens the effect of noise in the genetic expression data, and it makes the lowest rank representation matrix that is obtained by the sgLRR better for preserving the similarity among the cancer samples than the LLRR.
  In addition, the affinity matrix that is constructed based on the lowest rank representation matrix also plays a key role in the clustering. To briefly and clearly explain the contribution of the affinity matrix, we randomly select three typical datasets and give the heat maps based on matrix **Z**^∗^ and matrix **H**^∗^ for each respective dataset. The heat maps of the three selected datasets (CO-CH, CH-HN-CO and CO-CH-ES-HN) are as shown Fig. 6. In addition, in Fig. 6, the larger that the matrix element is, the brighter the corresponding position on the heat map. As we can see from Fig. 6, it is obvious that the grouping effect of matrix **H**^∗^ is better than that of **Z**^∗^.
4. In this part, we analyze the advantages of sgLRR method from the relationship between subspace number contained in datasets and method performance. According to Table 2, each of the six integrated datasets contains different types and amounts of cancer. In other word, the different integrated datasets contain different number of subspaces. And, there is reason to believe that the complexity of the internal geometric structures for the dataset has a notable positive correlation with the number of subspaces. Therefore, among the six integrated datasets, the CO-CH-ES-HN and ES-CO-PA-HN are the most complex, followed by the CH-HN-CO and ES-CH-HN that contain three types of cancer, and finally, followed by the integrated datasets CO-CH and PA-ES that contain two types of cancer. Based on the Table 3, we can see that the sgLRR method is better than the other methods as the subspace number increases. Specifically, the metrics of the sgLRR on the ES-CH-HN dataset are 2.92 (ACC), 30.14 (MCC), 4.03 (RI) and 26.37 (NMI) percentage points higher than those of the LLRR. Furthermore, for the CO-CH-ES-HN dataset, the percentages of the sgLRR are 1.69 (ACC), 13.66 (MCC), 1.92 (RI) and 5.85 (NMI) higher than those of the LLRR. This proves that the sgLRR method is more suitable for multi-cancer sample clustering than comparison methods.

**Table 3:** The clustering results of all methods on the different integrated datasets

Datasets	Measure	Method								
		K-means	T-SNE	LLE	NMF	PCA	LRR	LLRR	MLLRR	sgLRR
CO-CH	ACC	95.40	89.31	71.14	93.14	93.63	97.99	**99.66**	98.99	98.66
	MCC	80.06	70.21	88.58	75.53	72.99	46.76	91.06	90.78	**95.20**
	RI	91.43	49.85	63.66	87.18	88.07	96.04	**99.32**	98.00	97.34
	NMI	66.46	51.01	75.71	54.12	54.47	41.92	80.51	77.04	**87.51**
PA-ES	ACC	98.25	91.84	77.26	**99.16**	**99.16**	96.38	**99.16**	**99.16**	**99.16**
	MCC	84.73	83.74	62.04	**98.34**	**98.34**	77.98	96.71	96.71	**98.34**
	RI	97.37	84.97	66.46	**98.35**	98.33	93.00	98.34	98.34	98.34
	NMI	81.06	59.49	41.43	**93.86**	**93.86**	65.43	89.39	89.39	**93.86**
CH-HN-CO	ACC	89.22	83.03	80.99	76.99	85.77	95.86	97.99	84.77	**98.28**
	MCC	67.91	65.69	60.77	82.25	69.17	61.23	68.31	80.96	**87.25**
	RI	90.00	87.16	82.85	80.19	87.76	94.70	96.67	84.66	**97.48**
	NMI	73.55	78.43	69.07	76.22	72.59	68.25	73.81	77.83	**80.99**
ES-CH-HN	ACC	85.56	52.35	61.65	84.52	80.03	82.17	93.19	94.32	**96.11**
	MCC	66.26	32.62	42.01	67.44	66.08	43.04	61.18	66.49	**91.32**
	RI	82.67	60.97	63.89	80.25	78.15	72.36	89.07	90.30	**93.10**
	NMI	56.77	30.77	33.26	47.35	72.59	36.92	52.38	57.20	**78.75**
CO-CH-ES-HN	ACC	86.89	60.52	63.04	82.31	81.32	79.24	92.48	87.94	**94.17**
	MCC	70.00	65.30	79.30	51.78	71.30	52.44	78.05	73.03	**91.71**
	RI	89.43	74.59	71.98	85.07	86.57	81.58	91.42	88.95	**93.34**
	NMI	71.04	48.43	52.00	57.67	69.12	54.76	74.42	69.82	**80.27**
ES-CO-PA-HN	ACC	86.89	85.83	67.76	82.31	81.32	79.24	92.48	87.94	**94.17**
	MCC	79.51	78.52	77.40	84.23	**89.82**	59.21	88.38	83.63	85.49
	RI	89.43	91.45	78.06	85.07	86.57	81.58	91.42	88.95	**93.34**
	NMI	76.15	81.42	55.93	72.97	79.53	61.82	**81.24**	76.58	76.23

Note: The best clustering results are highlighted in bold

Through the above analysis, we can conclude that the combination of graph regularization based on manifold learning and the symmetry constraint plays a significant role in the sgLRR and achieves satisfactory results in multi-cancer samples clustering.

## Discussion

Based on the comparison and demonstration of the above experimental results, our proposed sgLRR method has advantages over other methods. The sgLRR method based on low rank representation has a great advantage in multi-subspace clustering. By means of symmetric constraint and sparse constraint, the influence of data noise on low-rank representation is alleviated. Meanwhile, the local geometric structure of data is retained through graph regularization constraint based on manifold learning, which improves the clustering effect in subspace clustering. Compared with other methods based on low rank representation, our method takes into account various factors that affect subspace clustering and improves the performance of the method. These advantages have been demonstrated in experiments with gene expression data from multiple cancers.

## Conclusions

In this paper, we introduce graph regularization based on manifold learning and symmetric sparse constraints into the original LRR and propose a novel method called the sgLRR. The original LRR method can capture the global geometrical information of the whole observation data. The lowest rank representation matrix **Z**^∗^ of the sgLRR method has the properties of the traditional LRR method and can capture the intrinsic local geometric information within data. In addition, the symmetry constraint weakens the effect of noise in the dictionary matrix and makes the lowest rank representation matrix **Z**^∗^ strictly and accurately preserve the similarity between samples.

We adopt the sgLRR method for multi-cancer samples clustering based on gene expression dataset. First, we use the sgLRR to obtain the lowest rank representation matrix **Z**^∗^. Then, based on the angular similarity information of the lowest rank representation matrix **Z**^∗^, we learn an affinity matrix **H** by using a unitary matrix that is obtained using skinny SVD. The results prove that the affinity matrix has a better grouping effect. Finally, based on the affinity matrix **H**, the spectral clustering algorithm (Ncuts) is used to obtain the clustering results.

We compare the experimental results from other methods to the sgLRR, including the K-means, T-SNE, LLE, NMF, PCA, LRR, LLRR and MLLRR methods. The experimental results show that the sgLRR method is a novel efficient method for multi-cancer sample clustering. The sgLRR method performs well on the dataset, which contain multiple subspaces. In future work, we will further study the sgLRR method. For example, the current method can be extended to identify characteristic cancer genes or to analyze cancer pathways.

## Data Availability

The datasets that support the findings of this study can be found in the [The Cancer Genome Atlas (TCGA)] https://cancergenome.nih.gov/.

## Abbreviations

ALM: The augmented Lagrange multiplier method;
LADMAP: The linearized adaptive direction method with the adaptive penalty method
LRR: Low-rank representation
Ncuts: Normalized cuts
sgLRR: The graph regularized low-rank representation under symmetric and sparse constraints method
SVD: Singular value decomposition
TCGA: The Cancer Genome Atlas
